# Epigenetics as Driver of Adaptation and Diversification in Microbial Eukaryotes

**DOI:** 10.3389/fgene.2021.642220

**Published:** 2021-03-16

**Authors:** Agnes K. M. Weiner, Laura A. Katz

**Affiliations:** ^1^Department of Biological Sciences, Smith College, Northampton, MA, United States; ^2^Program in Organismic and Evolutionary Biology, University of Massachusetts Amherst, Amherst, MA, United States

**Keywords:** epigenetics, adaptation, speciation, chromatin modification, non-protein-coding RNA, protists

## Introduction

Microbial eukaryotes, i.e., protists, represent the bulk of eukaryotic diversity in terms of species diversity and biomass. Protists are globally distributed in all ecosystems and play important roles in food webs and nutrient cycles. To date it remains enigmatic how protist diversity is generated, especially in lineages with large populations in ecosystems without apparent dispersal barriers (i.e., many marine species, species that encyst). We argue that epigenetic processes, such as chromatin modification and/or regulation by small non-protein-coding RNAs (npc-RNAs) that rapidly modify genomes and gene expression states, play important roles in driving phenotypic plasticity, differential adaptation and ultimately diversification of protists. Our argument is based on two recent developments in epigenetic research: (1) it is now clear that epigenetic processes were present in the last eukaryotic common ancestor (LECA) and are widespread across eukaryotes, and (2) numerous studies have demonstrated that at least some epigenetic marks can be inherited across generations. Given this, we suggest to combine morphometrics, genomics, and epigenomics for research on adaptability and diversification in microbial eukaryotes.

## Diversity of Microbial Eukaryotes

**Figure 1:**
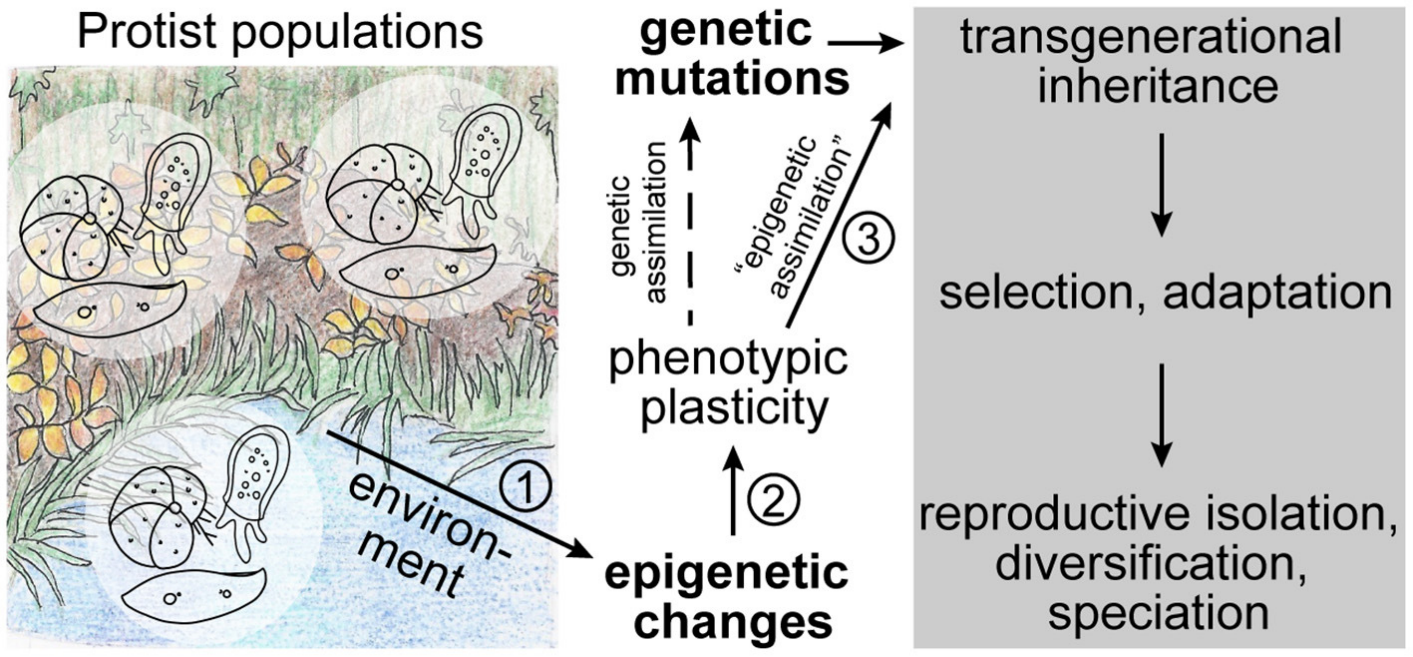
Theoretical sequence of events in ecological speciation driven by epigenetics. In addition to genetic mutations, naturally occurring protist populations experience epigenetic modifications that may be stochastic or be triggered by the environment. These modifications can lead to phenotypic plasticity in the population through changes in genome structure or gene expression states. If the epigenetic modification is followed by a genetic mutation, it may be fixed in the genome through genetic assimilation. However, if the epigenetic mark itself is stably inherited (i.e., “epigenetically assimilation”) across generations, it may represent a selectable advantage that can lead to an increase in fitness of the population and ultimately to adaptation, diversification, and speciation without changes to the genome. The numbers indicate the most critical steps in this sequence of events that we discuss throughout the text.

Many lineages of protists have a tremendous species diversity, which is reflected in a wide variety of morphologies and ecological functions (e.g., Adl et al., 2019). In addition, research of the last two decades has unearthed a large amount of cryptic diversity, suggesting a decoupling of morphological and molecular evolution (e.g., Katz et al., 2005; Šlapeta et al., 2005; Darling and Wade, 2008; Oliverio et al., 2014). Protists occur globally in all ecosystems, and while some species are endemic to certain areas, others have a cosmopolitan distribution and vast population sizes (e.g., Ryšánek et al., 2015; Faure et al., 2019). Large-scale barcoding studies revealed that some closely related cryptic species are able to co-occur in close biogeographical proximity (e.g., Amato et al., 2007; Weiner et al., 2014; Badger et al., 2017; Tucker et al., 2017). In addition, protists show a variety of complex life cycles, sometimes alternating sexual and asexual generations (e.g., Grell, 1973; Parfrey et al., 2008). Given these characteristics, the enormous species diversity is perhaps not surprising. However, which (molecular/epigenetic) mechanisms allow for speciation in microbes, especially in habitats with seemingly unlimited dispersal potential, remains unresolved. Several groups have hypothesized that differential adaptation to environmental factors may be the underlying driver for diversification in sympatry (e.g., Ryšánek et al., 2016; Irwin et al., 2017; Škaloud et al., 2019). However, for gene flow between populations to be overcome, mechanisms leading to the establishment of reproductive isolation would have to be fast and efficient. We argue that in order for rapid diversification to be achieved, epigenetic processes that regulate gene activity and that may be influenced by the environment play important roles in establishing phenotypic plasticity; if the epigenetic marks are inherited across generations—what we refer to as “epigenetic assimilation”—they can provide a fitness advantage to members of the population and ultimately lead to differential adaptation that drives speciation (Figure 1).

## Epigenetics in Microbial Eukaryotes

A prerequisite for our model of epigenetics as driver of ecological speciation in protists (Figure 1) to be valid is the widespread existence of epigenetic phenomena in microbial eukaryotes. Eukaryotic epigenetics comprises processes such as chromatin and DNA modifications and regulation by npc-RNAs (e.g., Razin and Riggs, 1980; Ng and Bird, 1999; Shabalina and Koonin, 2008), that are thought to have evolved originally for mediating genome conflict between mobile genetic elements and host genomes (Lisch, 2009; Fedoroff, 2012). The effects of epigenetics include, among others, gene activation or silencing, and altering genome structures through DNA elimination or polyploidization (e.g., Liu and Wendel, 2003; Bernstein and Allis, 2005). Most epigenetic research has focused on animals and plants, yet it was recently confirmed that the basic epigenetic gene toolkit was present in LECA and is now widespread throughout the eukaryotic tree of life (Aravind et al., 2014; Weiner et al., 2020). This highlights the importance of epigenetics for the functioning of eukaryotic genomes. For the majority of protists, however, knowledge on their epigenetics remains limited, mostly because many are uncultivable and annotated reference genomes are lacking. What is known so far mostly stems from research on model organisms, such as ciliates (Alveolata) and human pathogens [e.g., *Plasmodium* (Alveolata) and *Trypanosoma* (Excavata)].

In ciliates, which contain both a germline and somatic nucleus within one cell, epigenetics plays key roles in distinctions between the two genomes and in elimination of DNA during the development of a new somatic nucleus during reproduction (e.g., Chicoine and Allis, 1986; Jahn and Klobutcher, 2002; Chalker et al., 2013; Pilling et al., 2017). Small npc-RNAs, such as “scan RNAs” and “macronuclear RNAs,” bind to homologous regions in the genome or direct histone modifications (e.g., H3K9 methylation) in those regions to mark them for either retention or elimination (Chen et al., 2014; Swart et al., 2014). Similarly, npc-RNAs, so-called “template RNAs,” were found to be involved in the reordering of scrambled genes in some ciliates (e.g., Garnier et al., 2004; Nowacki et al., 2011). Another phenomenon of genome dynamics that is likely driven by epigenetics is the determination of ploidy levels throughout the life cycle. Many protist lineages, such as some Foraminifera (Rhizaria), ciliates and Amoebozoa have been observed to undergo significant changes in ploidy, sometimes containing thousands of copies of the genome that later are eliminated again (Parfrey et al., 2008; Bellec and Katz, 2012; Goodkov et al., 2020). In the case of ciliates, research suggested that RNA interference, which is part of the “epigenetic toolkit,” is driving these changes (Heyse et al., 2010).

In addition to these large-scale modifications to the genome architecture, epigenetic processes are involved in changes to the morphology or physiology of protists. This is especially prevalent in parasites, in which epigenetics controls virulence and cell differentiation through regulation of gene expression and thus plays an important role in host-pathogen interaction (e.g., Croken et al., 2012; Gomez-Diaz et al., 2012). For example, the formation of cysts (an important life cycle stage for host infection) in *Toxoplasma* (Apicomplexa), *Acanthamoeba* (Amoebozoa), and *Giardia* (Excavata) is driven at least partly by epigenetic mechanisms such as histone acetylation and methylation (e.g., H3K18 acetylation and H3R17 methylation in *Toxoplasma*; Saksouk et al., 2005; Dixon et al., 2010; Sonda et al., 2010; Moon et al., 2017; Lagunas-Rangel and Bermudez-Cruz, 2019). Antigenic variation, a strategy used by many pathogens (e.g., *Trypanosoma brucei, Giardia lamblia, Giardia doudenalis*, and *Plasmodium falciparum*) to avoid the host immune system, also is achieved through epigenetic regulation of gene expression (Kulakova et al., 2006; Elias and Faria, 2009; Juarez-Reyes and Castano, 2019; Lagunas-Rangel and Bermudez-Cruz, 2019). Their genomes contain many genes for surface proteins and the timing of gene expression is at least partly epigenetically regulated, e.g., through histone methylation (H3K4) or acetylation (H3K9) of the *var* genes in *Plasmodium falciparum* (e.g., Freitas-Junior et al., 2005; Guizetti and Scherf, 2013; Duffy et al., 2014). In this way, pathogenic protists are able to rapidly react and adapt to a changing host environment.

A further aspect of cell physiology that seems to involve epigenetics is mating type determination in ciliates (e.g., Pilling et al., 2017). While most ciliate species have different mating types, their number varies greatly (up to a 100; Phadke and Zufall, 2009) and so do the molecular mechanisms for mating type determination (e.g., Orias et al., 2017). For *Paramecium tetraurelia* it could be shown that the difference between its two mating types lies in the presence/absence of a transmembrane protein, whose expression is regulated by “scan RNAs” (Singh et al., 2014).

Despite the fact that details on the exact molecular processes and the genes/enzymes involved often remain scarce, the above-mentioned examples highlight the ubiquity and importance of epigenetics in the life histories of microbial eukaryotes.

## The Potential Role of Epigenetics in Driving Adaptation and Diversification

Ecological speciation through differential adaptation to environmental factors may be a plausible explanation for diversification in protists considering their often-large population sizes and wide biogeographic distribution. Here, we focus on the role of epigenetics in these events, yet we acknowledge that bottlenecks and drift likely are also important drivers of diversity in protists, especially in lineages with small populations and restricted distribution. However, the effects of these population genetic phenomena on epigenetics remain largely unknown.

In order to elucidate the interactions between ecology, epigenetics, and evolution that are the basis of our suggested model, special consideration has to be placed on the following questions (Figure 1): (1) does the environment trigger epigenetic variations, (2) can epigenetic modifications lead to phenotypic plasticity, and (3) are environmentally acquired epigenetic marks stably inherited to establish reproductive isolation and speciation? Over the last few years research efforts investigating these interactions have rapidly increased, yet so far mostly focusing on multicellular model species (e.g., Smith and Ritchie, 2013; Vogt, 2017; Boskovic and Rando, 2018; Perez and Lehner, 2019).

The notion that the environment influences epigenetic modifications is by now well-established. Many studies have focused on the effects of stress, toxin exposure or nutrition on epigenetic marks (e.g., Yaish et al., 2011; Collotta et al., 2013; Tiffon, 2018; Weyrich et al., 2019), and research on natural non-model systems showed epigenetic variability in populations across ecological gradients (e.g., Foust et al., 2016; Mcnew et al., 2017; Johnson and Kelly, 2020; Wogan et al., 2020). These studies usually focus on patterns of DNA methylation as this epigenetic modification is better understood and easy to analyze through bisulfite sequencing methods (e.g., Meissner et al., 2005; Smallwood et al., 2014). To our knowledge, few data exist on similar studies of protists, yet we argue that due to their ubiquitous occurrence across a wide range of environments, protist populations hold great promise for investigating environmental effects on epigenetic variation.

Elucidating the influence of epigenetics on phenotypic plasticity is more challenging as it can be difficult to rule out underlying genetic influences. However, recently progress has been made, mostly through experimental modifications to epigenetic marks on DNA or histones and the investigation of subsequent effects on the phenotype (e.g., Kronholm et al., 2016; Verhoeven et al., 2016). Research on a natural system was able to show that epigenetic modifications were more likely than genetic variability to have shaped the behavioral reproductive isolation in fish species (Smith et al., 2016). Similarly, epigenetic mechanisms were found to be responsible for phenotypic plasticity in asexual lineages allowing them to respond to environmental fluctuations (Castonguay and Angers, 2012). A further striking example of rapid phenotypic plasticity induced by epigenetics can be found in protist lineages that use antigenic variation through epigenetically regulated changes in gene expression to adjust to changing environments (see above).

For epigenetics to act as driver of speciation, it is important that epigenetic marks are stably inherited across generations, at least until reproductive isolation is established and/or genetic assimilation has occurred (Figure 1; discussed in Rey et al., 2016). The stable inheritance of epigenetic marks has long been debated as they were assumed to be eliminated during reproduction and only affect the current generation (discussed in: Richards, 2006), a view that is in line with the concepts of the modern synthesis and the rejection of the idea that acquired traits can be passed down to future generations (discussed in: Jablonka and Lamb, 2008; Bonduriansky, 2012). However, in recent years, examples of soft inheritance through transgenerational inheritance of epigenetic marks became more numerous (e.g., Richards, 2006; Bond and Finnegan, 2007; Perez and Lehner, 2019). Again, important examples can be found among protists, such as ciliates, in which acquired changes to the morphology or physiology, such as doublet morphology and mating types, are inherited to progeny without changes in the underlying nucleotide sequence (e.g., Pilling et al., 2017; Neeb and Nowacki, 2018). In addition, experimental evolution on the unicellular algae *Chlamydomonas* (Archaeplastida) showed that epigenetic variation is stably inherited across generations and thus influences adaptability of the organism (Kronholm et al., 2017).

## Conclusion

In recent years, a large amount of research has been published that focuses on the role of epigenetics in ecological speciation. It has been shown that environmentally induced epigenetic modifications can lead to differential gene expression and phenotypic plasticity. If these epigenetic marks are stably inherited across generations (“epigenetic assimilation”) and increase the fitness of the population, they could be substrate for selection and thus represent a first step toward ecological speciation (Figure 1).

While detailed information on the molecular processes of epigenetics in microbial eukaryotes remains scarce, its prominent role in shaping genome dynamics and driving phenotypic plasticity even across generations makes it likely that epigenetics is involved in generating their tremendous diversity. This, as well as their short generation times, make protists interesting model systems for studying the influence of epigenetics on adaptation and speciation. The model of ecological speciation driven by epigenetics presented here is consistent with the idea of rapid diversification in lineages with large population sizes and therefore weak genetic drift. Recent improvements in the sensitivity of high-throughput sequencing techniques to sequence the genomes, transcriptomes, and epigenomes of non-model microbes make this an exciting time to combine molecular, morphological, and epigenetic approaches for elucidating the origin of species diversity and a species' response to changing environmental conditions.

## Author Contributions

AW and LK developed the ideas and wrote the paper. All authors contributed to the article and approved the submitted version.

## Conflict of Interest

The authors declare that the research was conducted in the absence of any commercial or financial relationships that could be construed as a potential conflict of interest.
